# CONTRACT NURSE STAFFING AND NURSING HOMES: ANTECEDENTS AND CONSEQUENCES

**DOI:** 10.1093/geroni/igad104.0744

**Published:** 2023-12-21

**Authors:** Rohit Pradhan, Robert Weech-Maldonado, Jane Banaszak-Holl

## Abstract

Nursing home (NH) quality has been a matter of long-standing policy interest at the federal and state level, as it concerns the health and well-being of one of our most vulnerable populations. Nurse staffing intensity and skill mix have a consequential impact on NH care quality. Unfortunately, NH staffing has been marked by multitudes of challenges including high levels of turnover and absenteeism. In recent years, the utilization of agency or contract nurse staffing has increased as facilities face persistent staffing shortages exacerbated by COVID-19 which has had a disproportionate and devastating impact on NHs. The literature suggests that agency staffing negatively influences the performance of healthcare organizations due to multiple reasons including unfamiliarity with organizational culture, decline in teamwork, and the attendant high costs attached to contract staffing. However, contract nurse utilization practices and its impact on NH care has not been studied adequately, and much of the limited literature relies on datasets with limited understanding of agency staffing. The overarching goal of this symposium is to explore the antecedents and consequences of the increased utilization of contract nurse staffing. As part of the symposium, we include studies examining NH nursing skill mix and intensity, the organizational and market level factors associated with higher levels of contract staffing, and the impact of nurse contract staffing on NH staff turnover, quality, and financial performance. We believe that this series of studies would have important managerial and policy implications. This is a Research in Quality of Care Interest Group Sponsored Symposium.

